# Occupational burnout, self-compassion, and general health among dietitians in Türkiye: a cross-sectional study

**DOI:** 10.1186/s12913-026-15197-4

**Published:** 2026-07-25

**Authors:** Seher Aksakal Yasaroglu, Ziya Erokay Metin, Z. Begüm Kalyoncu Atasoy

**Affiliations:** 1 Department of Nutrition and Dietetics, Faculty of Health Sciences, Istanbul Aydin University, Istanbul, Türkiye; 2 Department of Nutrition and Dietetics, Gülhane Faculty of Health Sciences, University of Health Sciences, Ankara, Türkiye

**Keywords:** Occupational health, Occupational burnout, Dietitians, Self-compassion, Psychological distress

## Abstract

**Background:**

This study examined occupational burnout among dietitians in Türkiye and investigated whether self-compassion and general psychological health were associated with burnout levels. It also examined whether burnout differed across demographic and occupational subgroups.

**Methods:**

In this cross-sectional study, 382 actively practicing dietitians in Türkiye completed an online survey. Data collection instruments included the Maslach Burnout Inventory, Burnout Measure Short Version, Self-Compassion Scale, and General Health Questionnaire-12. Correlations, group comparisons, and regression analyses were performed to examine associations between burnout, self-compassion, psychological health, and occupational characteristics.

**Results:**

Higher burnout scores were significantly associated with lower self-compassion and poorer psychological health (*p* < 0.001). Higher self-compassion was associated with a more favorable burnout profile after adjustment for relevant covariates. Burnout also varied by occupational context. Dietitians working in the private sector and in foodservice/mass catering and research and development roles showed less favorable burnout-related patterns, including higher emotional exhaustion and depersonalization and lower personal accomplishment in relevant comparisons.

**Conclusion:**

Burnout among dietitians in Türkiye was associated with both occupational context and individual psychological factors. Higher self-compassion and better general psychological health were associated with lower burnout scores. These findings suggest that workplace conditions and psychological resources should be considered in future longitudinal research and intervention planning aimed at supporting dietitians’ occupational well-being and psychological resilience.

## Introduction

Occupational burnout is most commonly conceptualized according to Maslach’s model as a work-related psychological syndrome characterized by emotional exhaustion, depersonalization or cynicism, and reduced personal accomplishment or professional efficacy, arising in response to chronic occupational stress [1–3]. Burnout is especially prevalent among professionals engaged in emotionally demanding roles, such as those providing direct face-to-face services in health care [4, 5]. Physicians, nurses, and other allied health professionals are frequently exposed to high workloads, complex patient needs, and emotionally charged interactions, placing them at heightened risk for burnout [6–8]. Among these groups, dietitians represent a profession that has historically received less attention, despite their involvement in a wide range of responsibilities including individualized nutrition care, counseling, clinical decision-making, institutional food service management, etc., often carried out under significant time and resource constraints [9, 10].

Although the prevalence of burnout has been extensively documented in physicians and nurses [11], fewer studies have explored this phenomenon among dietitians [12, 13]. Dietitians experience moderate to high levels of burnout, particularly in relation to emotional exhaustion and reduced personal accomplishment, which may be influenced by role ambiguity, workload, and job insecurity [9, 12, 14]. This is particularly important given that dietitians frequently engage in emotionally demanding client interactions and must navigate complex nutritional care plans under time constraints [15]. Despite this, the number of empirical investigations focusing specifically on burnout among dietitians remains limited, particularly in terms of comparing its prevalence and contributing factors across different domains of dietetic practice, such as clinical nutrition, institutional food service, community nutrition, academia, and industry [12]. In parallel with growing interest in psychological resilience factors, self-compassion has been examined as a psychological resource associated with lower occupational stress and burnout [16, 17]. Understanding the role of self-compassion in dietitians could offer valuable insights into factors associated with professional well-being across various domains of dietetic practice.

Self-compassion is a psychological construct defined by treating oneself with kindness and understanding during times of failure or distress, recognizing shared human experiences, and maintaining balanced awareness of negative emotions [18, 19]. Research indicates that higher levels of self-compassion are associated with lower levels of stress, anxiety, and burnout across various healthcare professions, with recent findings showing associations between self-compassion and lower burnout scores, particularly emotional exhaustion and depersonalization, beyond the effects of demographic and occupational factors [17, 20, 21]. According to a systematic review by Crego et al., self-compassion has been consistently linked to benefits for mental health professionals, including lower burnout and compassion fatigue, improved well-being, and enhanced therapeutic effectiveness, highlighting the importance of fostering this trait in clinical practice. Despite these findings, the majority of existing studies have focused on physicians, nurses, and mental health professionals, with limited attention to dietitians [17].

In this context, this study sought to address these gaps by examining burnout levels among dietitians actively practicing in Türkiye. Since 1966, when the first graduates began working in the field, dietitians have played a critical role in healthcare and nutrition services [22]. In 1979, they were formally acknowledged as healthcare personnel through inclusion in the Civil Servants Law. Their roles and responsibilities in inpatient care institutions were defined in 1983, and in 2011, a dedicated professional law was enacted under Law No. 6225. The Turkish Dietetic Association, established in 1969, continues to serve as the primary professional body representing dietitians in the country [22]. As of July 2025, there is no additional registration or board examination required for dietitians to practice in Türkiye. Furthermore, the title “nutritionist” is not formally recognized in Türkiye; all professionals practicing in the field of nutrition and dietetics hold the title of “dietitian.” [23].

Given this historical and professional context, the present study aimed to assess burnout levels among actively practicing dietitians in Türkiye, examine the associations between burnout, self-compassion, and psychological distress as an indicator of general psychological health, identify demographic and occupational factors associated with burnout scores, and compare burnout-related scores across employment sectors and selected areas of dietetic practice. Based on previous research in healthcare professionals, the study hypothesized that higher self-compassion would be associated with lower burnout scores, higher psychological distress would be associated with higher burnout scores, and burnout-related scores would differ according to occupational context.

## Methods

This cross-sectional study was conducted to evaluate burnout levels among dietitians actively practicing in Türkiye and to examine demographic, occupational, and psychological variables associated with burnout. A cross-sectional design was selected because the aim was to describe burnout levels and examine their associations with psychological and occupational variables among actively practicing dietitians at a specific point in time, rather than to establish causal relationships or temporal directionality [24]. The target population consisted of actively practicing dietitians in Türkiye. At the time of study planning, the number of dietitians in Türkiye was estimated to be approximately 45,000 based on information obtained from the Turkish Dietetic Association (TDA). National higher education placement data also support the plausibility of this estimate, as 50,263 individuals graduated from Nutrition and Dietetics programs in Türkiye between 2014 and 2023 [25]; however, the TDA-based estimate was used for the primary sample size calculation. The required sample size was calculated using the finite population correction formula for estimating a population proportion, as described by Cochran [26, 27]. The calculation assumed a population size of 45,000, a 95% confidence level, a 5% margin of error, and an expected population proportion of 0.50, which provides the most conservative estimate when the true proportion is unknown. Based on these assumptions, the minimum required sample size was 381 participants. The final analyzed sample included 382 dietitians.

Participants were recruited using an online convenience sampling approach. The survey was administered through Google Forms and the survey link was distributed through multiple channels, including email, social media, and professional dietitian groups. The TDA supported recruitment by sending the survey invitation once to its members via email. Recruitment was not restricted to members of the TDA; any actively practicing dietitian in Türkiye who met the eligibility criteria could participate. Additional reminders were shared through social media and professional networks, but not through repeated TDA emails. The survey was administered anonymously and did not require participants to sign in with a Google account. Therefore, while responses were screened for completeness and obvious irregularities before analysis, duplicate participation could not be fully excluded. Because the survey link was disseminated through multiple online channels and could have been viewed or shared beyond the initial distribution channels, the total number of dietitians who received or viewed the invitation could not be determined. Therefore, a formal response rate could not be calculated.

The inclusion criteria were voluntary participation, being a graduate of a Department of Nutrition and Dietetics, and currently working as a dietitian in Türkiye. The exclusion criteria were not providing voluntary consent, not being a graduate of Nutrition and Dietetics, being unemployed, being retired, or pursuing another career path despite holding a Nutrition and Dietetics degree. Data collection was carried out through an online survey conducted from October to December 2024. The questionnaire consisted of “Sociodemographic Information Form”, “General Health Questionnaire-12 (GHQ-12)”, “The Burnout Measure Short Version (BMS)”, “Maslach Burnout Inventory (MBI)”, “Self-Compassion Scale (SCS)”.

This study was conducted in accordance with the principles of the Declaration of Helsinki. Prior to data collection, ethical approval was obtained from Istanbul Aydin University Non-Interventional Clinical Research Ethics Committee (Decision Number: 111/2024, 149/225). Participation in the study was entirely voluntary, and informed consent was obtained from all participants. The anonymity and confidentiality of the respondents were strictly maintained throughout the research process.

### Sociodemographic and dietetic profession data collection

In the first part of the questionnaire, participants were asked about age, sex, marital status, educational status, income, diagnosed health problems, smoking and alcohol use, and physical activity. Also, participants were asked about their university of graduation, year of graduation, postgraduate education status, fields of dietetic work since graduation, duration of employment, current field of work and sector (government, private or self-employment), and membership status in associations of the dietetic profession.

### Maslach Burnout Inventory (MBI)

The scale was developed by Maslach and Jackson in 1981 [28, 29] and adapted to Turkish by Ergin in 1993 [30–32]. It is one of the most widely used and extensively validated instruments for assessing burnout, with robust psychometric properties demonstrated across diverse occupational groups and cultural contexts [3, 33]. The scale assesses burnout across three dimensions: emotional exhaustion (9 items), depersonalization (5 items), and personal accomplishment (8 items), using a total of 22 items [30, 34, 35]. Each subscale score is calculated independently and interpreted separately, in accordance with the guidelines of the MBI manual. Specifically, it is not appropriate to sum the three subscale scores to produce a single total burnout score [36]. The emotional exhaustion and depersonalization subscales are scored such that higher scores indicate greater burnout. The personal accomplishment subscale was scored in its original direction and was not reverse-coded; therefore, higher scores indicate greater personal accomplishment, whereas lower scores indicate greater burnout-related reduction in personal accomplishment. Each MBI subscale was calculated and interpreted separately, and no total MBI score was computed. While the MBI is widely used to assess burnout symptoms, it is best understood as a spectrum-based measure rather than a diagnostic tool with universally agreed-upon cutoff scores or strict categorical boundaries [36, 37].

### The Burnout Measure Short version (BMS)

To address the need for a more practical tool than the original 21-item Burnout Measure (BM) developed by Pines and Aronson [38, 39], Pines introduced the Burnout Measure Short Version (BMS), a streamlined instrument with fewer items designed for easier use by researchers and practitioners [40, 41]. For this purpose, Pines adapted the 10-item short form (BMS) of the 21-item scale. The BMS was created to assess individuals’ levels of physical, emotional and mental fatigue, and the 10 selected items were determined in line with the contextual basis of the BM. The scale is assessed on a seven-point system (1: Never, 7: Always) and is self-reported to measure the level of occupational burnout. To calculate an individual’s burnout score, the total score from all 10 items is divided by 10; the resulting average represents their level of occupational burnout. An increase in BMS scores corresponds to higher degrees of burnout, signifying more pronounced physical, emotional, and cognitive fatigue. Analyses of data from different ethnicities, occupations and student groups showed that the internal consistency coefficients of the scale ranged from 0.85 to 0.92 [42, 43].

### Self-Compassion Scale (SCS)

Self-Compassion Scale (SCS) was developed by Neff [44, 45] and the Turkish validity and reliability study of the scale conducted by Akın et al. [46, 47]. This scale consists of 26 items and includes six sub-dimensions: self-compassion, self-judgment, awareness of sharing, isolation, consciousness, and overidentification. The scale uses a five-point Likert-type rating; (1) Never, (2) Rarely, (3) Often, (4) Usually, and (5) Always. The scale provides both a total self-compassion score and individual subscale scores. Items belonging to the negative subscales; (i) self-judgment, (ii) isolation, and (iii) over-identification is reverse coded. The total self-compassion score is calculated by first computing the mean of each subscale and then averaging all six subscale means. In the evaluation of the total score, scores between 1 and 2.5 indicate low self-compassion, scores between 2.5 and 3.5 indicate moderate self-compassion and scores between 3.5 and 5.0 indicate high self-compassion [46, 48].

### General Health Questionnaire-12 (GHQ-12)

The General Health Survey-12 (GHQ-12) is a self-administered questionnaire developed by David Goldberg and used to assess mental status in the community and in primary health care services. This questionnaire is widely used in various studies because the questions are short and understandable, the administration time is short, validity studies have been conducted and it has high sensitivity and specificity [49, 50].

The GHQ-12 is an assessment tool consisting of 12 questions in total. These questions ask about the participant’s symptoms over the last few weeks and each question consists of four options: “never”, “as often as usual”, “more often than usual”, and “very often”. Responses to the questionnaire questions are scored; options “a” and “b” are given a score of “0” and options “c” and ‘d’ are given a score of “1”. The total score thus ranges from 0 to 12, with higher scores indicating poorer mental health and greater psychological distress. This tool detects a wide range of psychological disorders, particularly those on the anxiety–depression spectrum.

The GHQ-12 has been shown to be valid and reliable in Turkish populations. The Turkish adaptation and psychometric validation were conducted by Kilic et al., demonstrating good internal consistency and construct validity. Factor analysis of the Turkish version supports a two-factor structure; (i) anxiety/depression, and (ii) social dysfunction [51, 52].

### Statistical analysis

Normality of continuous variables was assessed using the Shapiro-Wilk and Kolmogorov-Smirnov tests [53]. For comparisons between two groups, the Mann-Whitney U test was used for variables that were not normally distributed, and the independent samples t-test was used for normally distributed variables. For comparisons involving three or more groups, the Kruskal-Wallis H test was used for variables that were not normally distributed, with post hoc comparisons performed using Dunn’s test [54]. Relationships between categorical variables were examined using Pearson’s chi-square test, Yates correction, Fisher’s exact test, or Fisher’s exact test with Monte Carlo correction, as appropriate. Relationships between non-normally distributed quantitative variables were analyzed using Spearman’s rho correlation coefficient [54].

Multiple linear regression was used to examine factors associated with the BMS score, as this outcome met the assumptions required for standard linear regression. Robust regression models were fitted separately for the MBI subscales, namely emotional exhaustion, depersonalization, and personal accomplishment, because these outcomes did not fully meet the assumptions of ordinary least squares regression, including normality of residuals, homoscedasticity, and absence of influential outliers. Robust regression was performed using the MASS package, and the confidence intervals reported for the robust regression models were based on robust standard errors. Variables included in the regression models were selected based on theoretical relevance, previous literature on burnout among healthcare professionals, and their relevance to the study objectives, rather than solely on bivariate statistical significance. Model assumptions were assessed by examining residual distributions and residual plots. Multicollinearity was evaluated using variance inflation factors (VIF), and all VIF values were below 10, indicating no serious multicollinearity. For multiple comparisons in Table 2, Bonferroni correction was applied where appropriate to reduce the risk of type I error arising from multiple testing [55, 56].

Quantitative data are presented as mean±standard deviation (SD) and median (minimum-maximum), while categorical data are presented as frequencies and percentages. Missing data were handled using complete-case analysis; participants with missing values on variables required for a given analysis were excluded from that analysis. A p-value of < 0.05 was considered statistically significant. Data analyses were performed using IBM SPSS Statistics version 25.0 [57] and R software [58]. During the preparation of this work, the authors used ChatGPT-4o to improve the readability of the manuscript. After using this tool, the authors reviewed and edited the content as needed and take full responsibility for the content of the publication.

## Results

### Dietitian characteristics

A total of 382 dietitians participated in the study (Table 1). The mean age of participants was 28.97 ± 6.08 years (range: 22–62), and most participants were female (90.1%). Most participants were relatively recent graduates, with a mean time since graduation of 7.34 ± 5.95 years (median: 6, range: 1–43 years). The mean BMI was 22.83 ± 3.68 kg/m². Among participants who reported having children, the mean number of children was 1.28 ± 0.59. Regarding household income, 58.4% of participants reported that their income was either more than adequate or adequate to meet monthly expenses without difficulty, whereas 41.6% reported some level of financial strain. More than half of the participants were self-employed (52.1%), followed by those working in the private sector (32.5%) and public sector (15.5%). In terms of professional affiliation, 49.5% reported being members of the Turkish Dietetic Association, while 82.2% reported following the association on social media.

**Table 1:** General characteristics and scales of the dietitians participating in the study (*n* = 382)

	Mean ± SD^1^	Median (Min-max)/%
**Age**	28.97 ± 6.08	27 (22–62)
Number of children*	1.28 ± 0.59	1 (1–4)
**Time Since Graduation**	7.34 ± 5.95	6 (1–43)
**BMI**	22.83 ± 3.68	22.032 (15.432–36.934)
**Scales/Questionnaires**		
General Health Questionnaire	5.416 ± 4.178	5 (0–12)
The Burnout Measure Short Version	4.02 ± 1.37	4.1 (1–7)
Emotional exhaustion	15.293 ± 7.301	16 (0–34)
Depersonalization	5.418 ± 3.635	5 (0–17)
Personal accomplishment	21.170 ± 4.408	21 (0–32)
Self-Compassion Scale	3.04 ± 0.68	3 (1.25–4.8)
	**Frequency**	**Percentage**
**Sex**		
Female	344	90.1
Male	38	9.9
**Marital status**		
Married or living with partner	165	43.2
Single	207	54.2
Divorced	10	2.6
**Presence of children**	85	22.3
**Monthly household income**		
More than adequate to comfortably cover monthly expenses	107	28.0
Adequate to meet monthly needs without difficulty	116	30.4
Sufficient for basic needs, but financial strain is present	114	29.8
Insufficient to meet basic needs	45	11.8
**Smoking status**		
No	266	69.6
Regularly	58	15.2
Occasionally	58	15.2
**Alcohol consumption status**		
No	236	61.8
Regularly	7	1.8
Occasionally	139	36.4
**Exercise habits**		
No	73	19.1
Regularly	103	27.0
Occasionally	206	53.9
**Presence of chronic disease**	101	26.4
**Turkish Dietetic Association membership status**	189	49.5
**Turkish Dietetic Association Social Media Following Status**	314	82.2
**Current employment sector**		
Self-employed	199	52.1
Private sector	124	32.5
Public sector	59	15.5

*Number of children was calculated among participants who reported having children

Table 2 presents scale scores according to current field of work. Current field of work was assessed as a multiple-response variable; therefore, the categories were not mutually exclusive, and the same participant could contribute to more than one work-field category. Accordingly, these comparisons should be interpreted as exploratory descriptive comparisons rather than comparisons between fully independent groups.

**Table 2:** Comparison of scale scores according to current field of work selected as multiple-response categories

	Clinical nutrition	Academia	Online dietetics	Public health nutrition	Sports Nutrition	Foodservice/ Mass catering	Nutrition Support Team	*R*&D Dietitian	Overall *p* value
**Postgraduate work experience (n, %)**									
Less than 1 year	55 (18.5%)	49 (72.1)	63 (20.6)	46 (46.5)	64 (47.1)	72 (39.8)	48 (50)	48 (78.7)	> 0.05
1–3 years	87 (29.2)	9 (13.2)	111 (36.3)	27 (27.3)	38 (27.9)	59 (32.6)	23 (24)	6 (9.8)	
3–5 years	66 (22.2)	5 (7.4)	73 (23.9)	7 (7.1)	17 (12.5)	15 (8.3)	8 (8.3)	1 (1.6)	
More than 5 years	90 (30.2)	5 (7.4)	59 (19.3)	19 (19.2)	17 (12.5)	35 (19.3)	17 (17.7)	6 (9.8)	
**General Health Questionnaire**									
Total score	5.05	4.82	5.58	5.59	4.59	5.73	4.58	6.60	> 0.05
**The Burnout Measure Short Version**									
Total Score	3.90	4.05	4.03	4.08	3.67	4.19	4.18	4.14	> 0.05
**Maslach Burnout Inventory**									
Emotional Exhaustion	14.83	16.53	14.47	15.24	13.63	17.29	17.95	18.20	> 0.05
Depersonalization	5.06^a^	6.29^ab^	5.25^a^	5.24^ab^	4.93^ab^	6.82^b^	6.05^ab^	8.20^ab^	**< 0.05**
Personal Accomplishment	21.76^ab^	20.88^ab^	21.53^ab^	20.66^ab^	22.87^a^	20.30^b^	21.63^ab^	19.20^ab^	**< 0.05**
**Self-Compassion Scale**									
Total Score	3.10	2.85	3.02	3.02	3.20	3.01	3.14	3.20	> 0.05
Self-kindness	2.84	2.66	2.74	2.68	2.91	2.69	2.76	2.84	> 0.05
Self-judgment	2.63	2.67	2.71	2.59	2.53	2.68	2.58	2.36	> 0.05
Common Humanity	2.91	2.69	2.83	2.80	2.93	2.79	3.03	2.70	> 0.05
Isolation	2.73	3.04	2.82	2.71	2.56	2.75	2.66	2.50	> 0.05
Mindfulness	2.96	2.56	2.86	2.72	3.09	2.88	3.00	2.85	> 0.05
Over-identification	2.74	3.10	2.79	2.77	2.62	2.86	2.72	2.30	> 0.05

Values are presented as mean unless otherwise stated. Postgraduate work experience is presented as n (%). Current field of work was assessed as a multiple-response variable; therefore, categories are not mutually exclusive and the same participant could contribute to more than one column. Accordingly, comparisons across work-field categories should be interpreted as exploratory descriptive comparisons rather than comparisons between fully independent groups. Different superscript letters indicate statistically significant pairwise differences after post hoc adjustment; groups sharing the same letter do not differ significantly. *p* < 0.05 was considered statistically significant

Participants showed variability in psychological distress, burnout, and self-compassion scores. The GHQ-12 scores spanned the full possible range, indicating variability in psychological distress across the sample. In the exploratory descriptive comparisons by current field of work, GHQ-12 scores were numerically highest among dietitians working in R&D (6.60) and foodservice/mass catering (5.73), followed by public health nutrition (5.59) and online dietetics (5.58). The lowest mean GHQ-12 scores were observed among those working in nutrition support teams (4.58) and sports nutrition (4.59); however, the overall comparison was not statistically significant. Similarly, BMS scores showed numerical variation across work fields, with the highest mean scores observed among dietitians working in foodservice/mass catering (4.19), nutrition support teams (4.18), and R&D roles (4.14), although the overall comparison was not statistically significant. Emotional exhaustion scores were also numerically higher among those working in R&D (18.20), nutrition support teams (17.95), and foodservice/mass catering (17.29), but this difference was not statistically significant. Statistically significant differences were observed for depersonalization and personal accomplishment. Depersonalization scores were highest in R&D (8.20) and foodservice/mass catering (6.82), with post hoc comparisons indicating significantly higher scores in foodservice/mass catering than in clinical nutrition and online dietetics. Personal accomplishment scores, where higher scores indicate greater personal accomplishment, were lowest among R&D dietitians (19.20) and those working in foodservice/mass catering (20.30), and highest among those working in sports nutrition (22.87). Post hoc comparisons indicated that sports nutrition had significantly higher personal accomplishment scores than foodservice/mass catering.

Internal consistency analyses were conducted for the measurement tools in the present sample. Cronbach’s alpha values were 0.908 for the GHQ-12, 0.934 for the Burnout Measure Short Version, and 0.949 for the Self-Compassion Scale total score. For the Maslach Burnout Inventory, Cronbach’s alpha values were 0.897 for emotional exhaustion, 0.771 for depersonalization, and 0.775 for personal accomplishment. For the Self-Compassion Scale subscales, Cronbach’s alpha values were 0.853 for self-kindness, 0.875 for self-judgment, 0.755 for common humanity, 0.816 for isolation, 0.843 for mindfulness, and 0.811 for over-identification. These values indicated acceptable to high internal consistency for the scales and subscales used in the present study.

### Burnout and self-compassion measures of dietitians

As shown in Table 3, multiple regression analysis indicated that higher levels of psychological distress (GHQ-12) and lower levels of self-compassion were significantly associated with higher burnout scores (BMS) among dietitians. Higher self-compassion was independently associated with lower BMS scores (β = -0.316, *p* < 0.001), whereas higher psychological distress was associated with higher BMS scores (β = 0.462, *p* < 0.001). Fewer years since graduation were also associated with higher BMS scores (β = -0.200, *p* = 0.046).

**Table 3:** Multiple linear regression analysis examining factors associated with Burnout Measure Short version scores among dietitians (*N* = 382)

	β0 (%95 CI)	S. Error	β1	Test statistic	*p*	VIF
Age	0.019 (-0.021–0.058)	0.020	0.097	0.939	0.349	5.688
Time Since Graduation	-0.039 (-0.077 - -0.001)	0.019	-0.200	-2.012	**0.046**	5.238
Self-Compassion Scale – Total Score	-0.614 (-0.821 - -0.408)	0.105	-0.316	-5.880	**< 0.001**	1.532
General Health Questionnaire – Total Score	0.153 (0.118–0.188)	0.018	0.462	8.671	**< 0.001**	1.505
Sex (Ref: Female)						
Male	-0.346 (-0.752–0.061)	0.206	-0.088	-1.680	0.095	1.444
Marital Status (Ref: Couple habitation)						
Single	-1.011 (-1.931 - -0.091)	0.465	-0.105	-2.172	**0.032**	1.244
Divorced	-1.108 (-2.237–0.022)	0.571	-0.094	-1.939	0.055	1.258
Spouse’s employment status (Ref: Employed)						
Unemployed	-0.345 (-0.811–0.122)	0.236	-0.079	-1.459	0.147	1.568
Presence of children (Ref: None)						
Yes	-0.084 (-0.383–0.214)	0.151	-0.032	-0.558	0.578	1.789
Monthly household income (Ref: Income level 2^2^)						
Income level 4^4^	-0.55 (-0.91 - -0.19)	0.182	-0.197	-3.020	**0.003**	2.263
Income level 3	-0.189 (-0.516–0.138)	0.166	-0.069	-1.141	0.256	1.946
Income level 1^1^	0.368 (-0.129–0.865)	0.251	0.079	1.465	0.145	1.540
Smoking status (Ref: None)						
Regularly	0.266 (-0.14–0.672)	0.205	0.061	1.293	0.198	1.196
Occasionally	0.069 (-0.265–0.403)	0.169	0.021	0.408	0.684	1.348
Alcohol consumption status (Ref: None)						
Regularly	0.658 (-0.417–1.734)	0.544	0.056	1.210	0.228	1.141
Occasionally	0.545 (0.282–0.809)	0.133	0.199	4.094	**< 0.001**	1.255
Exercise habits (Ref: None)						
Regularly	0.051 (-0.312–0.414)	0.184	0.0174	0.28	0.780	2.045
Occasionally	-0.111 (-0.415–0.192)	0.154	-0.0428	-0.725	0.470	1.849
Presence of chronic disease (Ref: No)						
Yes	0.319 (0.047–0.591)	0.138	0.108	2.321	**0.022**	1.157
BMI^5^	0.034 (-0.001–0.069)	0.018	0.098	1.894	0.060	1.427
Current employment sector (Ref: Self-employed)						
Private sector	0.465 (0.155–0.775)	0.157	0.153	2.963	**0.004**	1.411
Public sector	0.059 (-0.263–0.382)	0.163	0.0192	0.364	0.717	1.486
TDA^6^ membership status (Ref: No)						
Yes	-0.234 (-0.482–0.014)	0.125	-0.089	-1.868	0.064	1.211
Work schedule (Ref: Full time)						
Part time (*n* = 88)	-0.058 (-0.379–0.264)	0.163	-0.018	-0.355	0.723	1.294

F:16.105; *p* < 0.001; R^2^ =%72.86, β0: Unstandardized beta coefficient, β1: Standardized beta coefficient, VIF: Variance Inflation Factor, ^1^Income level 1: Insufficient to meet basic needs, ^2^Income level 2: Sufficient for basic needs, but financial strain is present, ^3^Income level 3: Adequate to meet monthly needs without difficulty, ^4^Income level 4: More than adequate to comfortably cover monthly expenses, BMI: Body Mass Index, ^6^TDA: Turkish Dietetic Association, Ref: Reference

Compared with participants reporting sufficient income for basic needs but with financial strain, those reporting more than adequate income had significantly lower BMS scores (β = -0.197, *p* = 0.003). Private sector employment was associated with higher BMS scores compared with self-employment (β = 0.153, *p* = 0.004). Single marital status was associated with lower BMS scores compared with being married or living with a partner (β = -0.105, *p* = 0.032). Occasional alcohol consumption was associated with higher BMS scores compared with no alcohol consumption (β = 0.199, *p* < 0.001), and presence of chronic disease was associated with higher BMS scores (β = 0.108, *p* = 0.022). Sex, parenthood, spouse’s employment status, BMI, smoking status, exercise habits, public sector employment, TDA membership, and part-time work schedule were not significantly associated with BMS scores in the overall model. The final model explained 72.9% of the variance in BMS scores (F = 16.105, *p* < 0.001).

Robust regression models were constructed to examine factors associated with the three MBI dimensions (Table 4). Higher self-compassion was significantly associated with lower emotional exhaustion and depersonalization scores, and with higher personal accomplishment scores (*p* < 0.001 for all). This pattern indicates that higher self-compassion was associated with more favorable scores across all three MBI dimensions. Working in the private sector was associated with higher emotional exhaustion and depersonalization scores and lower personal accomplishment scores, indicating a less favorable burnout profile in this employment setting. Psychological distress (GHQ-12) was significantly and positively associated with emotional exhaustion (*p* < 0.001), but was not significantly associated with depersonalization or personal accomplishment. Presence of children was significantly associated with lower depersonalization scores, but not with emotional exhaustion or personal accomplishment. Spouse unemployment, compared with spouse employment, was significantly associated with lower emotional exhaustion and depersonalization scores, but not with personal accomplishment. Time since graduation, TDA membership status, work schedule, and BMI were not significantly associated with any of the three MBI subscales in the robust regression models. The overall explained variance was 54.0% for emotional exhaustion, 43.3% for depersonalization, and 37.4% for personal accomplishment. Thus, among the three MBI dimensions, emotional exhaustion showed the highest explained variance and was significantly associated with several variables, including self-compassion, psychological distress, private sector employment, and spouse employment status.

**Table 4:** Robust regression models for factors associated with Maslach Burnout inventory subscale scores among dietitians (*N* = 382)^1^

**Variable**	Model 1 (Emotional Exhaustion)			Model 2 (Depersonalization)			Model 3 (Personal Accomplishment)		
	β0(%95 CI)	SE	*P* value	β0(%95 CI)	SE	*P* value	β0(%95 CI)	SE	*P* value
Time Since Graduation	-0.141 (-0.396–0.114)	0.129	0.276	-0.029 (-0.171–0.114)	0.072	0.693	0.115 (-0.056–0.285)	0.086	0.187
Self-Compassion Scale – Total Score	-3.256 (-4.599 - -1.912)	0.680	**< 0.001**	-2.243 (-3.006 - -1.479)	0.386	**< 0.001**	2.272 (1.354–3.189)	0.464	**< 0.001**
General Health Questionnaire – Total Score	0.509 (0.277–0.741)	0.117	**< 0.001**	0.028 (-0.106–0.162)	0.068	0.679	0.094 (-0.065–0.253)	0.081	0.245
Spouse’s unemployment (Ref: spouse employed)	-3.541 (-6.632 - -0.45)	1.564	**0.025**	-2.181 (-3.925 - -0.436)	0.882	**0.015**	1.452 (-0.654–3.559)	1.066	0.175
Presence of children (Ref: None)	-1.888 (-3.87–0.095)	1.003	0.062	-1.205 (-2.331 - -0.078)	0.570	**0.036**	0.678 (-0.664–2.02)	0.679	0.320
Current employment sector in private institutions	5.034 (2.963–7.105)	1.048	**< 0.001**	1.776 (0.598–2.954)	0.596	**0.003**	-1.999 (-3.405 - -0.594)	0.711	**0.006**
TDA^2^ membership status (Ref: No)	-1.183 (-2.817–0.452)	0.827	0.155	-0.147 (-1.081–0.787)	0.472	0.756	0.89 (-0.229–2.009)	0.566	0.118
Work schedule (Ref: Full time)	0.157 (-1.957–2.27)	1.069	0.884	-0.135 (-1.33–1.06)	0.604	0.824	-1.243 (-2.69–0.204)	0.732	0.092
BMI^3^	-0.138 (-0.368–0.092)	0.116	0.238	-0.09 (-0.221–0.041)	0.066	0.176	0.125 (-0.031–0.28)	0.079	0.115
R^2^	54%			43.3%			37.38%		
F for change in R^2^	7.24; (*p* < 0.001)			4.531; *p* < 0.001			3.581; (*p* < 0.001)		

^1^All models are adjusted for age, sex, marital status, presence of chronic disease, income, smoking, alcohol consumption, and exercise

^2^Turkish Dietetic Association, ^3^Body Mass Index

## Discussion

This study addressed a critical gap in the literature by examining burnout among dietitians in Türkiye, a professional group often overlooked in burnout research. While burnout is well-documented in other healthcare professions (28, 29), the psychological and occupational correlates of burnout in dietitians, particularly the role of self-compassion, remain underexplored [9, 12]. Using validated assessment tools, this study investigated the associations between burnout, self-compassion, psychological distress, and key demographic and occupational factors.

The main findings of this study highlight both individual and occupational influences on burnout among dietitians. Less favorable burnout-related scores across the BMS and MBI dimensions were associated with lower self-compassion and greater psychological distress, measured with GHQ-12. Importantly, higher self-compassion was consistently associated with a more favorable burnout profile, including lower emotional exhaustion and depersonalization scores and higher personal accomplishment scores. Among these, emotional exhaustion showed the strongest associations with self-compassion and psychological distress, indicating its central role in the burnout experience. Differences also emerged across employment sectors and work settings: dietitians employed in the private sector reported significantly higher burnout scores compared to those who were self-employed, and depersonalization scores were notably elevated among those working in foodservice/mass catering and R&D dietetics. These findings underscore the importance of considering both individual psychological traits and workplace context in understanding burnout-related patterns among dietitians.

Dietitians manage a wide range of responsibilities, from nutritional assessment and counseling to administrative tasks and interprofessional collaboration, all of which can increase their mental workload [59–61]. Frequently shifting between these diverse roles may heighten stress levels and may be associated with greater burnout-related strain [12, 62]. A recent meta-analysis confirmed that burnout prevalence among dietitians and nutritionists is high and comparable to rates observed in other health professionals [12]. Moreover, burnout levels among dietitians appear to be significantly shaped by work context, with higher rates observed in those managing high-risk clinical workloads or with longer professional experience [10, 12, 62]. Consistent with these observations, the findings revealed notable variation in burnout scores among dietitians in Türkiye, with some participants reporting levels of emotional exhaustion and depersonalization indicative of occupational strain. However, the overall levels of burnout were not markedly high, suggesting that while burnout symptoms are present within this population, they may not be as severe or widespread as in other high-risk healthcare professions. While standardized thresholds for categorizing burnout severity remain debated, the distribution of scores across validated instruments indicates a meaningful but not extreme burden of burnout symptoms in this professional group. Notably higher depersonalization scores were observed among those working in foodservice/mass catering and R&D dietetics, suggesting that specific fields may present distinct emotional challenges. These findings highlight the importance of implementing structured support systems, particularly for dietitians working in emotionally demanding specialties such as oncology or intensive care. Initiatives like regular debriefing meetings, peer mentorship programs, and resilience workshops that are potentially organized in collaboration with professional associations could play a vital role in helping dietitians navigate the unique emotional strains of their work [60, 63–65].

In the present study, dietitians working in the private sector reported significantly higher burnout levels compared to those in the public sector and self-employed participants. This finding suggests that the private sector may pose unique occupational stressors for dietitians, potentially related to job insecurity, performance pressures, limited institutional support, or rigid productivity expectations. Employment precarity may also be relevant in dietetics, particularly for early-career professionals. Recent research on dietetics graduates has described the transition into employment as an uncertain process, with some graduates facing difficulty obtaining dietetics-related work and insecure or precarious employment arrangements, including short-term, casual, or contractor roles [66, 67]. While the present study did not directly measure job insecurity or contract type, these factors may help contextualize the association between private-sector employment and less favorable burnout-related scores. A recent cross-sectional study from Türkiye similarly highlighted the relevance of employment-related factors in dietitians’ occupational well-being, reporting associations between career decision regret, job satisfaction, perceived income level, workplace institution, and burnout-related scores among dietitians [68]. Although the literature on sectoral differences in burnout is mixed, the present findings align with more recent evidence suggesting that private sector professionals may be particularly vulnerable to burnout. Yeh et al. (2018) reported elevated emotional exhaustion among private compared to public employees in Taiwan, especially in smaller organizations [69], while more recent evidence has also linked precarious employment and psychosocial work conditions with burnout and minor mental disorders [70]. Similar concerns have been reported in physiotherapy, where occupational pressures, perfectionism, and moral injury were associated with burnout among practising physiotherapists [71]. Recent evidence from healthcare settings also suggests that private-sector work may involve specific psychosocial risks, including time pressure, administrative burden, scheduling conflicts, and variable access to wellbeing resources, all of which may be associated with less favorable burnout-related patterns [72]. In the current study, private-sector dietitians similarly exhibited a less favorable burnout profile, with higher emotional exhaustion and depersonalization and lower personal accomplishment levels than their public-sector and self-employed peers, underscoring that occupational context and organizational support infrastructures play a crucial role in shaping burnout-related patterns in dietetic practice.

In addition, a significant proportion of nutrition and dietetics graduates face delayed employment, which may contribute to burnout even before active practice begins. For example, a U.S. study reported that 11% of recent graduate dietitians were still job-seeking six months after degree completion, with 2% remaining unemployed at the 12-month mark [66]. Unemployment and underemployment may be associated with financial stress, reduced self-efficacy, and disruption of professional identity, all of which may be linked to psychological distress and burnout [73]. Although unemployment was not directly assessed in the present sample of actively practicing dietitians, this broader labor-market context may help explain why financial strain and career-stage variables were relevant to burnout. In the present study, lower household income and fewer years since graduation were associated with higher BMS scores, suggesting that economic insecurity and early-career conditions may be associated with higher burnout scores among dietitians. Future studies should examine employment instability, underemployment, job insecurity, and career-development pressures more directly in relation to burnout among nutrition and dietetics graduates and practicing dietitians. Professional bodies and employers may also consider whether structured career guidance, mentorship, and early-career support could help address burnout vulnerability, although such interventions require evaluation in longitudinal or intervention studies.

In Türkiye, dietitians are legally recognized within the “Health Services” class alongside physicians, nurses, and other professionals [74]. However, professional recognition, role clarity and perceived value within multidisciplinary teams may still vary across work settings [75, 76]. Although these factors were not directly measured in the present study, they may be relevant to understanding burnout-related dimensions such as depersonalization and personal accomplishment among dietitians. Previous research has suggested that dietitians may experience challenges related to professional identity, role recognition and interprofessional collaboration [9, 77, 78]. Future qualitative and longitudinal studies should examine how role clarity, professional recognition, workload, administrative burden, and team-based working conditions contribute to burnout experiences among dietitians in different employment sectors and practice areas.

A growing body of international research has examined self-compassion as a psychological resource associated with lower burnout among healthcare professionals [17, 79]. The present findings are consistent with this literature within the Turkish context: dietitians reporting higher self-compassion consistently exhibited a more favorable burnout profile, with lower emotional exhaustion and depersonalization and higher personal accomplishment. Notably, this association was observed after adjustment for demographic and occupational variables, aligning with previous studies in which self-compassion has been associated with lower emotional strain [17, 74]. These converging findings point to the potential value of examining self-compassion-oriented approaches in professional development and workplace wellness programs. Institutional support for reflective practice, peer support, or self-compassion-oriented training may be a promising focus for future longitudinal and intervention studies examining burnout prevention and occupational well-being [77].

A major strength of this study is that it is one of the few investigations to examine the interrelationships between burnout, self-compassion, and general psychological health specifically among dietitians. The focus on dietitians in Türkiye addresses an underexplored professional group within the burnout literature. The use of two established burnout measures, the Maslach Burnout Inventory and the Burnout Measure Short Version, together with validated measures of self-compassion and psychological distress, also strengthens the assessment of the main constructs and supports comparability with previous studies. In addition, the sample included dietitians working across different employment sectors and practice areas, allowing burnout-related patterns to be examined across a range of occupational contexts.

However, several limitations should be acknowledged. First, the study used a cross-sectional design; therefore, causal relationships or temporal directionality between burnout, self-compassion, psychological distress, and occupational factors cannot be inferred [80]. Second, participants were recruited through a voluntary online survey distributed via email, social media, professional dietitian groups, and support from the Turkish Dietetic Association. Because the total number of dietitians who received or viewed the invitation could not be determined, a formal response rate could not be calculated. This recruitment approach may also have introduced self-selection bias, as dietitians with a particular interest in burnout or psychological well-being may have been more likely to participate [81]. Third, the sample may underrepresent dietitians who are not connected to professional networks, Turkish Dietetic Association communication channels, or online professional groups. It may also underrepresent those experiencing the highest levels of occupational distress, who may have been less likely to complete the survey. Fourth, all data were self-reported, which may have introduced recall bias, reporting bias, and social desirability bias [82–84]. Fifth, current field of work was assessed as a multiple-response variable; therefore, work-field categories were not mutually exclusive, and the same participant could contribute to more than one category. Comparisons across work-field categories should therefore be interpreted as exploratory descriptive comparisons rather than comparisons between fully independent groups. Finally, cultural, regulatory, and labor-market conditions specific to Türkiye may limit the generalizability of the findings to dietitians working in other countries or healthcare systems. Future longitudinal, qualitative, and cross-national studies are needed to clarify directionality, examine contextual mechanisms, and inform interventions tailored to different dietetic practice settings [85].

## Conclusion

Overall, the results were consistent with the study hypotheses that higher self-compassion and better general psychological health were associated with lower burnout scores among practicing dietitians in Türkiye, and these associations remained evident across multiple burnout dimensions after adjustment for demographic and occupational variables. Burnout-related scores also varied by employment sector and selected areas of practice, with less favorable patterns observed particularly among dietitians working in the private sector. In the exploratory comparisons by practice area, foodservice/mass catering and R&D roles showed numerically less favorable burnout-related profiles, including higher depersonalization and lower personal accomplishment scores, although work-field comparisons should be interpreted cautiously because categories were overlapping multiple-response groups. These findings suggest that both workplace conditions and individual psychological resources should be considered in future research and intervention planning. Although causal conclusions cannot be drawn from the cross-sectional design, the results point to the need for longitudinal studies to clarify directionality and to examine whether workplace-level strategies and self-compassion-oriented approaches can support dietitians’ occupational well-being. 

## Data Availability

The data used and/or analysed during the current study are available from the corresponding author on reasonable request.
